# Should all MCI with Alzheimer’s biological diagnosis receive anti-amyloid therapy?

**DOI:** 10.1038/s41419-026-08456-z

**Published:** 2026-02-10

**Authors:** Paolo Maria Rossini, Chiara Pappalettera

**Affiliations:** Department of Neuroscience and Neurorehabilitation, IRCCS San Raffaele Roma, Rome, Italy

**Keywords:** Alzheimer's disease, Neurophysiology, Predictive markers

## Abstract

Our perspective addresses one of the most pressing and timely debates in contemporary neurology and health policy: whether the recent approval of anti-amyloid monoclonal antibodies for Alzheimer’s disease should extend to all individuals with mild cognitive impairment (MCI; a large population of tens of millions of individuals worldwide mainly represented in Countries with aged population) who test positive for amyloid biomarkers, despite wide variability in prognosis and therapeutic response and the epidemiological demonstration that only about half of them manifest symptoms of dementia. The manuscript highlights three central themes. First, while epidemiological and meta-analytic data confirm that MCI significantly increases the risk of dementia, more than half of affected individuals—many of whom are biomarker-positive for amyloid/tau—do not progress to dementia even over long- term follow-up. Second, recently approved anti-amyloid therapies, although representing a landmark in disease-modifying treatments, carry high costs, non-negligible risks (particularly amyloid-related imaging abnormalities), and uncertain long-term real-world benefits. Third, indiscriminate prescription of these agents risks exposing large numbers of subjects to unnecessary harm while placing unsustainable burdens on healthcare systems. We argue that the field should urgently move to identify and validate accurate and sustainable instruments for risk-stratified treatment pathways, integrating genetic, clinical, neuropsychological, neuroimaging, and fluid biomarker data including risk and resilience factors to refine prognostication. In addition, we call on the scientific community, journals, and policymakers to foster dialog that bridges neurology, geriatrics, bioethics, health economics, and patient advocacy, so that clinical innovation is matched by ethical responsibility and equitable implementation.

## Introduction

Mild cognitive impairment (MCI) is an intermediate stage between physiological cognitive ageing and pathological decline of cognition leading to dementia. It affects tens of millions of individuals worldwide (mainly in aged Countries); it is frequently the prodromal phase of Alzheimer’s disease (AD)-associated dementia, but not inevitably so [1]. Individuals with MCI typically demonstrate abnormalities on one (single-domain) or more (multi-domain) level-II neuropsychological tests. Nevertheless, they maintain independence in all activities of daily living, including those requiring high levels of cognitive ability [1, 2]. This functional preservation distinguishes MCI from dementia and has significant implications for public health strategies, including prevention policy and treatment decisions.

Despite this frame, the recommendations of the International Working Group (IWG) and Alzheimer’s Association (AA) agree that MCI with a biological diagnosis represents symptomatic AD. In particular, in the revised IWG framework [3], AD is defined as a clinical–biological construct, requiring both biomarker evidence of AD pathology and the presence of corresponding clinical symptoms. Individuals who are cognitively normal but biomarker-positive are therefore described as “asymptomatic at risk for AD.” MCI subjects with biomarker-positive are defined as “prodromal AD”.

Additionally, the 2024 AA criteria [4] define AD as a biological continuum, in which the presence of β-amyloid and tau pathology constitutes “Alzheimer’s disease” irrespective of the clinical stage. Within this framework, asymptomatic individuals with both biomarkers are classified as being in the preclinical stage of AD.

Both group of Experts converge on the view that MCI individuals with biomarker evidence of Alzheimer’s pathology represent symptomatic stages of the disease—designated prodromal Alzheimer’s disease by the IWG and MCI due to Alzheimer’s disease by the AA criteria.

In our opinion, while the 2024 AA criteria define Alzheimer’s disease purely on a biological basis—anchored in the presence of β-amyloid and tau pathology—this conceptualization may blur the boundary between pathological process and disease manifestation. The biological framework assumes that biomarker evidence of AD pathology necessarily indicates the presence of the disease, even in asymptomatic individuals. However, mounting evidence suggests that this equivalence is often not valid with important implications for the use of anti-amyloid monoclonal antibodies.

First, the presence of amyloid and tau pathology in cognitively normal individuals does not invariably predict clinical decline. Longitudinal cohort studies have shown that a significant subset of biomarker-positive older adults remain cognitively intact for many years, suggesting individual differences in resilience or cognitive reserve (e.g., due to neural compensation, education, genetic factors, or lifestyle) [5–7]. From this perspective, biological abnormality does not automatically translate into disease, as the organism may maintain functional integrity despite pathological burden.

Second, defining Alzheimer’s disease purely by biomarkers risks pathologizing normal aging and medicalizing risk rather than identifying illness. The IWG 2024 recommendations explicitly attempt to avoid this by maintaining a clinical-biological definition: the label “Alzheimer’s disease” applies only when both biological and clinical criteria are met, while biomarker-positive asymptomatic individuals are described as “at risk of AD.” This distinction preserves the conceptual separation between pathophysiological process and disease state.

Finally, considering resilience highlights that the trajectory from biological abnormality to symptomatic disease is probabilistic, not deterministic. Integrating resilience factors into disease definitions could yield a more dynamic model—where pathology, reserve, and clinical expression interact—rather than a strictly biomarker-based dichotomy.

### MCI natural history and prognostic uncertainty

Follow-up studies—as also seen in recent literature meta-analysis- consistently report that 30–50% of individuals with MCI will progress to dementia over 3–5 years [8]. Indeed, a recent meta-analysis of 66 clinical studies investigated the progression from mild cognitive impairment (MCI) to all-cause dementia. The pooled cumulative conversion rate was 41.5% (95% CI: 38.3–44.7%; *I*² = 95.5%), and no outliers were identified among the included studies. The overall annual conversion rate (ACR) was 10.9% (95% CI: 9.7–12.1%; *I*² = 98.4%), with rates varying according to follow-up duration. Specifically, the ACR was 13.1% (95% CI: 11.1–15.2%) in studies with a 3-year follow-up, 11.5% (95% CI: 10.1–13.0%) in studies with 3.1 to 5 years of follow-up, and 7.8% (95% CI: 6.7–9.0%) in studies with follow-up periods exceeding 5 years. Of the included studies, 20 had a 3-year follow-up, 26 had follow-up durations between 3.1 and 4.9 years, and 20 had follow-up periods of 5 years or longer [8]. However, it should be noted that these results refer to unselected MCI cohorts, without stratification based on Alzheimer’s disease biomarkers.

In fact, MCI individuals with biomarker-confirmed Alzheimer’s disease pathology (A+ or A+T+) exhibit higher rates of progression, as shown in several recent longitudinal and meta-analytic studies [9–14].

Evidence from large multicohort and prospective studies shows that while abnormal AD biomarkers substantially increase the likelihood of progression, a sizeable proportion of biomarker-positive MCI individuals remain clinically stable over 2–3 years. In the largest analysis to date, Vos et al. (2015) [12] examined 1607 MCI participants from 13 cohorts and reported that the 3-year conversion rate was 50% in IWG-1 prodromal AD, 61% in IWG-2 prodromal AD, and 59% in the NIA-AA high-AD-likelihood (A+T+) group—meaning that 41–50% of biomarker-positive individuals did not progress within 3 years. Consistent findings emerge across other cohorts. In Wolk et al. [9], among 232 MCI participants, amyloid-positive individuals showed a 53.6% conversion rate at 36 months, indicating that 46.4% remained stable. In Parnetti et al. [15], survival analysis showed that 81% of MCI with a low Aβ₁₋₄₂/p-tau ratio progressed, leaving 19% non-converters at 3 years. Moon et al. found that among amyloid-positive MCI, conversion at 2 years ranged from 19.7% (without depression) to 40.8% (with depression). Okello et al. [16] reported that 47% of PiB-positive MCI converted within 1 year, whereas others remained stable for up to 3 years. In Iaccarino et al. (2017) [17], overall conversion at ≈2.2 years was 46.7%, while individuals positive for both FDG-PET and PiB-PET showed 100% progression during follow-up.

Finally, in our last work [18] accepted for publication in Alzheimer’s and Dementia: TRCI, analyzing the Interceptor cohort, among 178 amyloid-positive MCI participants, 107 did not convert, corresponding to a percentage of ~60% over three years. Taken together, these stratified data indicate variable results but at the same time that even within biomarker-positive MCI—whether defined by PET, CSF, or multimodal criteria—between 40% and 50% of individuals typically remain clinically stable over 2–3 years [5, 19]*.* This divergence likely reflects the influence of protective and resilience factors, including cognitive reserve, genetic modifiers beyond APOE, lifestyle factors, and perhaps unrecognized neuropathological heterogeneity*.* The implication is clear: biomarker positivity alone, besides heralding higher risk of developing dementia, does not inevitably equate to clinical progression. The distinction between “at risk” and “destined to develop disease” is central to the current debate on whether anti-amyloid interventions—particularly those carrying high direct/indirect costs and the risks for significant side effects- should be deployed in all eligible MCI patients

### The advent of anti-amyloid disease-modifying therapies

Over the past three years, major regulatory agencies—including the US Food and Drug Administration (FDA), the European Medicines Agency (EMA), the Japanese Pharmaceuticals and Medical Devices Agency (PMDA), the Chinese National Medical Products Administration (NMPA), and the UK Medicines and Healthcare products Regulatory Agency (MHRA)—have approved monoclonal antibodies targeting brain amyloid-beta, such as Lecanemab and Donanemab. These agents are the first to receive disease-modifying claims in Alzheimer’s disease, representing a watershed moment in neurodegenerative therapeutics. The FDA granted accelerated approval for Lecanemab in January 2023 and converted it to traditional approval in July 2023 [20]; Japan followed with approval in September 2023 [21]*,* China in January 2024 [22], the UK in August 2024 [23] and the EMA issued a positive opinion in late 2024 with final authorization in 2025 [24], albeit with restrictions excluding APOE ε4 homozygotes. Donanemab has similarly been approved in the US (2024), Japan (September 2024), the UK (October 2024), and most recently received a positive CHMP opinion at the EMA in July 2025.

Indications across agencies typically focus on early symptomatic stages—mild cognitive impairment (MCI) due to AD or mild AD dementia—with confirmed amyloid pathology, and with genetic stratification excluding APOE ε4 homozygotes in several regulatory frameworks, given their higher risk of amyloid-related imaging abnormalities (ARIA) such as cerebral edema and microhemorrhages. The therapeutic rationale is compelling: intervene before extensive synaptic loss, network organization degradation and neuronal death, when the brain retains sufficient structural and functional reserve to maintain everyday function. If disease progression can be slowed at this point, years of preserved independence could be gained. Unfortunately, until now, the approved drugs have a cost/benefits ratio which discourage their use on the whole MCI population without any pre-selection. Early post-approval experience indicates a cautious but expanding clinical uptake. In Japan, around 6000 patients received treatment during the first year after launch, with structured MRI monitoring and infusion-capacity programs highlighting significant logistical and safety challenges [21]. Such requirements substantially affect feasibility and costs, particularly in health systems with limited infusion or neuroimaging infrastructure.

Economic evaluations remain heterogeneous: a Canadian model estimated an incremental cost-effectiveness ratio (ICER) of about CAD 62,800 (~€44,000) per QALY [25], whereas a Swedish analysis identified a cost-effective annual price near SEK 33,900 given modest QALY gains and substantial non-drug costs [26]. Conversely, health technology assessments, including draft NICE guidance in the UK, have concluded that, at current pricing and monitoring requirements, lecanemab and donanemab are unlikely to be cost-effective in publicly funded healthcare systems.

At the same time, it is also true that the economic landscape of Alzheimer’s disease diagnosis is changing rapidly with the introduction of blood-based biomarkers (BBMs), which hold the potential to streamline and reduce at least the cost of diagnostic pathways. Recent evidence demonstrates that BBMs measuring plasma Aβ42/40, phosphorylated tau (p-tau181, p-tau217, p-tau231), and neurofilament light chain show high concordance with CSF and PET measures of amyloid and tau pathology, enabling earlier, less invasive, and more cost-efficient screening [27–29]. Several health-economic and consensus studies further suggest that incorporating BBMs could substantially reduce reliance on PET and CSF testing, especially in the initial diagnostic triage, thereby mitigating the overall economic impact of large-scale diagnostic implementation [30–32].

### Implementation challenges

Despite this promise, real-world deployment of unselected amyloid-positive MCI subjects’ treatment with antiamyloid monoclonal antibodies faces several practical and ethical hurdles:

1. *Exclusion criteria*—The stringent exclusion criteria used in trials select for an almost idealized population, patients with minimal comorbidities, no anticoagulant use, and low frailty level [33–35], which is rarely encountered in clinical practice. Furthermore, patients receiving anti-amyloid therapy who experience acute ischemic stroke may be ineligible for intravenous thrombolysis [36, 37].
2. *Mode of administration and acute monitoring needs*—These agents are delivered by intravenous infusion every two to four weeks, requiring healthcare infrastructure for administration and short-term observation for infusion reactions.
3. *Risk of amyloid-related imaging abnormalities (ARIA)*—ARIA, including vasogenic edema and microhemorrhages, is a well-documented class effect, necessitating regular brain MRI during the first year of treatment. The incidence and severity are significantly greater in APOE ε4 homozygotes, who are therefore excluded in many protocols.
4. *Economic burden*—Beyond the high direct drug cost, mandatory pre-treatment investigations (amyloid PET or lumbar puncture), genetic testing, repeated MRI, infusion services, and clinical follow-up all add to the financial footprint. Indirect costs, such as patient and caregiver time, further strain healthcare systems.
5. *Modest short-term effects and uncertain long-term benefit*—While trial data demonstrate modest slowing of cognitive decline over 18 months, it remains unknown whether these effects translate into meaningful delay of functional dependence in diverse, real-world populations.

### The ethical dilemma

If only 30–50% of MCI patients progress to dementia within 5 years and longer follow-up, even when amyloid-positive, then a substantial proportion—perhaps half—of treated individuals may never develop clinically significant disease. For them, treatment would offer no benefit, yet they would still bear the risks, burdens, and costs of therapy.

This raises several ethical and health policy questions:

- Is it justifiable to treat all biomarker-positive, non-APOE ε4 homozygote MCI patients pre-emptively, given this uncertainty?
- Should healthcare systems bear the high cost of widespread treatment without more precise prognostic tools?
- What degree of risk–benefit asymmetry is acceptable for preventive therapy in neurodegeneration, especially in the absence of imminent functional decline?

Balancing individual autonomy with the responsible stewardship of finite healthcare resources remains one of the most enduring and complex challenges in modern medicine. On the one hand, policies that are overly restrictive may prevent certain at-risk individuals from accessing potentially beneficial interventions, thereby undermining patient-centered care and limiting opportunities for improved health outcomes. On the other hand, policies that are overly permissive may extend access too broadly, exposing large numbers of individuals to unnecessary risks, interventions of limited efficacy, or harms that outweigh potential benefits, while simultaneously straining healthcare systems already operating under resource constraints.

Additionally, several studies indicate that the majority of older individuals with AD pathology also harbor additional neurodegenerative or vascular processes, which can attenuate or obscure the clinical effects of targeted anti-amyloid therapies if treatment is initiated in later stages [38–41]. At the same time, earlier intervention, although biologically more plausible, carries the opposite risk of overtreating individuals who may remain clinically stable for years or never progress to dementia, owing to individual resilience, compensatory mechanisms, or protective comorbid profiles. Achieving the right balance requires careful consideration of clinical evidence, ethical principles, and societal priorities, as well as ongoing dialog between patients, clinicians, researchers, policymakers, and the broader community. Ultimately, the goal is to create frameworks that safeguard equity and justice while respecting individual choice and ensuring that scarce healthcare resources are deployed in ways that maximize benefit and minimize harm.

### Toward risk-stratified treatment pathways

In response to these concerns, national health systems are exploring strategies to improve patient selection [42–44]. Notably, the EPAD program established a large biomarker-rich longitudinal cohort to identify high-risk individuals and test stratification workflows [45]. The AMYPAD project evaluated how amyloid-PET informs diagnostic triage and management in real-world settings [46]. National memory-clinic networks such as MEMENTO/BALTAZAR in France have implemented standardized clinical, CSF, MRI, and PET profiling to support data-driven risk stratification [47]. Finally, public consortia are leveraging AI-based models to refine prognostic risk tiering [48].

Moreover, in these years, several nomogram-based and artificial intelligence (AI)-driven frameworks have demonstrated promising accuracy and clinical applicability.

Nomogram-based models combining MRI, CSF, and cognitive variables have shown excellent predictive accuracy, with high AUCs reaching 0.9, while large cohort-based risk scores using routinely collected clinical data demonstrate more pragmatic but still robust discrimination (C-statistics ≈ 0.7–0.8) [49–54].

Artificial intelligence (AI) and deep-learning frameworks have further enhanced predictive performance and generalizability, identifying high-risk subgroups and dynamic trajectories of decline across heterogeneous MCI populations [55–57].

Together, these and several companion studies demonstrate that multidimensional prognostic algorithms— whether based on traditional nomograms or AI-driven models—can achieve predictive accuracy (AUC ≈ 0.7–0.9) and offer clinically interpretable frameworks for individualized risk stratification. The goal is to move beyond binary biomarker positivity toward multi-parameter prognostic models that incorporate risk/resilience factors, including socio-demographic, neuropsychological, clinical, genetic, neuroimaging, fluid biomarker and lifestyle variables. Ideally, such models would enable stratification of MCI patients into high-, intermediate-, and low-risk groups for progression to dementia.

The development of such tools faces its own challenges:

- The need for longitudinal datasets, including risk/resilience factors, with harmonized procedures and multimodal data collection.
- Validation across diverse populations and healthcare settings.
- Integration into clinical workflows in a time- and cost-efficient manner.
- Ethical handling of prognostic information in asymptomatic or minimally impaired individuals.

Non-invasive, scalable, and affordable screening methods—such as blood-based biomarkers for amyloid and tau—are likely to play a pivotal role. Several are already in advanced stages of validation, though widespread clinical adoption is pending regulatory and reimbursement frameworks [20].

### The role of the scientific community and journals

Scientific journals can accelerate progress by prioritizing studies that focus on developing, validating, and implementing such stratification tools. A dedicated publication track for research in this space could encourage cross-disciplinary collaboration between neurology, geriatrics, primary care, epidemiologists, neuroimaging, data science, and health economics. Equally important is the inclusion of bioethics, regulatory science, and patient advocacy in this dialog, since questions of access, fairness, and acceptability cannot be separated from the scientific and clinical debates.

Ultimately, the debate over anti-amyloid therapy in MCI is not just a clinical question—it is a societal one. It touches on the boundaries of preventive and personalized medicine, the acceptable thresholds of uncertainty, and the responsibilities of health systems in allocating resources where the line between benefit and futility is thin. The decision to treat individuals who are still functionally independent with drugs that carry measurable risks, uncertain long-term benefits, and significant financial implications requires a societal consensus, not just expert opinion.

In this context, journals can serve as platforms for balanced debate, publishing not only trial results but also real-world implementation studies, cost-effectiveness analyses, and patient-reported outcome research. Only by integrating these diverse perspectives can the field move toward a framework that is robust, transparent, and adaptable to the rapid evolution of therapeutics in Alzheimer’s disease.

By fostering dialog between clinicians, researchers, policymakers, and patients/caregivers, we may move toward a consensus framework that maximizes benefit, minimizes harm, and ensures that the promise of disease-modifying therapy in Alzheimer’s disease is realized in a way that is both clinically sound and ethically justifiable. Such a framework should be dynamic, revisited as new evidence emerges, and sensitive to the heterogeneity of health systems across the globe. In this way, the scientific community can help ensure that these therapies are not only a triumph of biomedical innovation, but also an example of responsible, equitable, and patient-centered medicine.
